# Construction and Evaluation of Rainwater Harvesting System for Domestic Use in a Remote and Rural Area of Khulna, Bangladesh

**DOI:** 10.1155/2014/751952

**Published:** 2014-09-14

**Authors:** Biplob Kumar Biswas, Bablu Hira Mandal

**Affiliations:** Department of Chemical Engineering, Jessore University of Science and Technology, Jessore 7408, Bangladesh

## Abstract

Scarcity of pure drinking water during the dry season (November–March) is a major problem in Bangladesh, which needs to be addressed. This crisis has been further aggravated due to surging populations. Rainwater can provide some of the cleanest naturally occurring water and can hold a great potential in dealing with the current challenge of acute arsenic poisoning as well as physical water scarcity in many parts of Bangladesh. In this connection, rainwater harvesting (RWH) system has been constructed in a very remote and rural village in Khulna, Bangladesh, for a 4-membered household. It consists of a concrete catchment of 40 m^2^ area, a supporting and collection system made of PVC pipes, and two locally available plastic storage tanks having capacity of 2000 L each. The study also investigates the quality aspects of the stored rainwater, which include measurement of pH, alkalinity, hardness, total dissolved solids (TDS), iron, chloride, nitrate, and turbidity, using standard methods. The results showed that not only the quality of harvested rainwater is good but also the amount of water is enough for a 4-membered household to meet its domestic use throughout the year.

## 1. Introduction

Water that covers about 70% of earth's surface is an essential substance for the nature and the ecosystem of the world. It has a number of unique chemical and physical properties that make it indispensable to life and, in fact, it makes up about 60% of adult body weight [1, 2]. Water is reported to be grouped into atmospheric, surface, and ground water where atmospheric water includes moisture contained in the cloud, which precipitates as snow and rain [3]. Rain water, on the other hand, is a form of precipitation in which liquid water falls to the earth's surface [3]. Rainwater and snowmelt are thought to be the primary sources of all drinking water in this world [4].

About 97.5% of all water on earth is salt water, leaving only 2.5% as fresh water, which can be found in various forms such as glaciers and permafrost and groundwater and surface as well as atmospheric water [5, 6]. So it is evident that fresh water, though renewable, is a limited resource. Reports revealed that 768 million people worldwide lack access to safe water, which is often termed as physical water scarcity [7], whereas 1.8 billion people are predicted to live in regions with absolute water scarcity by 2025 [8]. This has happened due to unplanned management of water resources, insufficient planning, and insufficient political will. Water scarcity is, therefore, thought to be a serious problem throughout the world and mitigating this problem is one of the biggest challenges of the 21st century [9]. The Millennium Development Goal's (MDG) target 7C calls for reducing by half the proportion of the population without sustainable access to safe drinking water and basic sanitation by 2015 [10].

Lack of access to safe drinking water is an increasing problem in the southwestern coastal areas of Bangladesh, where salinity in ground and surface water and arsenic as well as iron contamination of shallow aquifers are supposed to be the two major concerns for this. Such problems are considered to be significant barriers to improving community health and reducing poverty. In pursuit of having drinking water, people often rely on fresh water pond, which is normally preserved by the local community. But nowadays, freshwater ponds are also not available due to horizontal saline intrusion in the coastal area. Therefore, people, particularly women and girls, are to travel up to several kilometers for collecting water for drinking, which means that a significant number of productive hours are consumed. Moreover, this has impacts on women's safety, girls' education, health risks, and microeconomy of individual family. However, after the hit of Cyclone-Sidr in November 2007 and Cyclone-Aila in May 2009, all water sources became dysfunctional and sanitation facilities were either destroyed or damaged by tidal surge in the coastal areas of Bangladesh. Therefore, the existing fresh water sources, both surface and groundwater, became contaminated by either salinity or bacteria [11].

Drinking water is generally obtained from two sources: ground water (wells, boreholes, etc.) and surface water (rivers, lakes, etc.). However, these sources account for only 40% of total precipitation as depicted by WaterAid [12]. Thus it is evident that there are considerable scopes for the collection of rainwater when it falls. The potential of rainwater harvesting (RWH) and the urgency of mitigating water-scarcity related problems are also confirmed by the fact that many researchers are working on these issues. RWH is a simple technique of collecting rainwater instantly making sure that it does not run off into river or stream or does not soak into the ground or does not become contaminated [4, 13]. Such technique has been practiced for centuries. However, a low-cost and easy-to-maintain water supply system in difficult geohydrological areas is very much in demand to ensure a sustainable development. In this regard, RWH can be considered as a probable solution of drinking water crisis in arsenic affected areas, saline zone in the coastal areas, and areas prone to groundwater depletion. Ghisi et al. showed a huge potential of potable water savings and preservation of water resources through RWH in as many as 62 cities in Brazil [14]. Rainwater is free from salinity as well as arsenic contamination and is safe too if it is maintained hygienically. The physical, chemical, and bacteriological characteristics of harvested rainwater usually represent a suitable and acceptable standard of potable water. Harvested rainwater can be used not only in drinking purposes but also in cooking, washing, and bathing. The main limitation of this option is nonavailability of rain water around the year. But it can be widely used as supplementary source if rainwater is properly stored in rainy season.

The geographic location of Bangladesh is very unique having the Bay of Bengal and the Indian Ocean to the south and the Himalayas to the north. Because of such physical location, a tropical monsoon climate prevails in Bangladesh that has made it one of the wettest countries of the world. Therefore, climate of Bangladesh is characterized by difference in wind pattern and variation in rainfall (with high rainfall from April to September). The mean annual rainfall in Bangladesh is about 2320 mm [15] while that in Khulna district is about 1800 mm [16]. Theoretically 20% of the total rainfall might satisfy almost the whole of Dhaka city's demand, collected during the monsoon [17]. Based on such data and information it can be predicted that rainfall that occurred in Khulna area could well satisfy the demand of a small household of a rural area, Bajua near Sundarban, of Khulna district in Bangladesh if it is efficiently harvested. Therefore, the objectives of this study are to construct a RWH system for domestic use in a small household of a rural area (Bajua) of Khulna, to evaluate the effectiveness of the rainwater harvesting system, and to monitor the quality (physical as well as chemical) of harvested rainwater as well as to assess the acceptability of rainwater use for domestic purpose.

## 2. Materials and Methods

### 2.1. The Research Site

**Figure 1:**
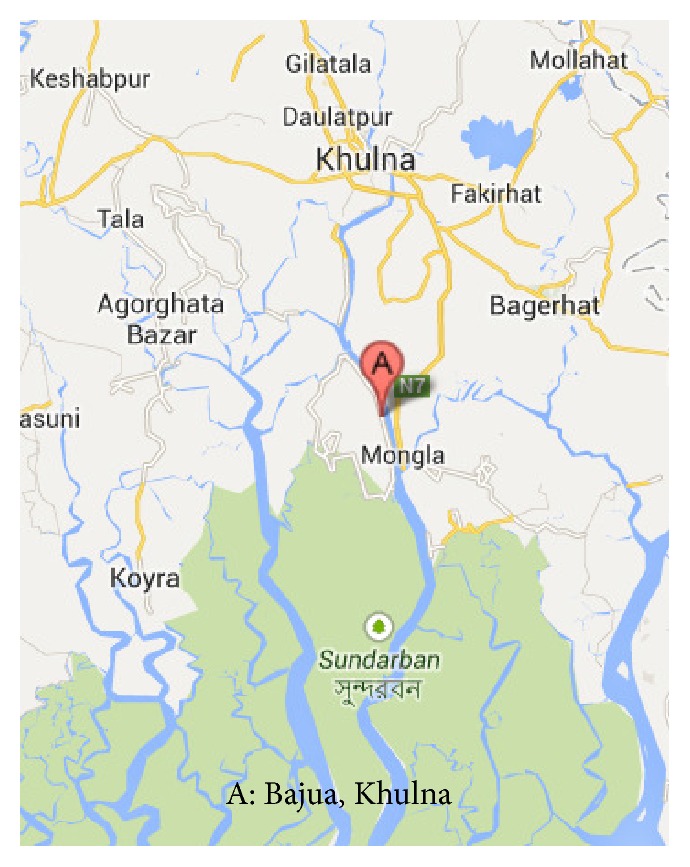
Location of the research site [18].

The research site, Bajua, a rural village of Khulna district in Bangladesh, is situated at the southern part of Khulna district and is nearly 46 km away from Khulna Metropolitan area. Bajua is selected as the study area because the area has extensive scarcity of pure drinking water. It is located at 22°35′′ latitude and 89°569′′ longitude and is near the Sundarban, one of the famous world heritage site declared by UNESCO. A location map is shown in Figure 1 [18]. As mentioned above the mean annual rainfall in this area is about 1800 mm [16].

### 2.2. Methodology

The study was conducted through some sequential steps, which were necessary to abide by for obtaining scientific outcome. The methodology of this study was confined to in-depth field observation, 20 years mean rainfall data analyses (1989–2008), calculation of water demand per capita of the studied household, collection, storage, and usage of harvested water of that household, and laboratory tests. The field observation showed that the studied locality has a severe problem of shortage of pure drinking water, which is instigated for selecting the study area. However, the study was conducted during the period from April 2011 to September 2012. Rainfall data for a period of 20 years (1989–2008) was collected from Bangladesh Agricultural Research Council [16].

A RWH system was constructed by using locally available components. The RWH system used in this study was composed of three basic components: roof catchment, supporting collection system (gutter, screen/roof washer, and flushing system), and storage tank. Harvested water was tested in laboratory.

### 2.3. Estimating Domestic Water Demand

Estimating domestic water demand, in reality, is not so easy. Children and adults use different amounts of water and seasonal water use varies, with more water being used in the summer season. The number of household members staying at home may also vary at different times of the year because during religious as well as local cultural festivals relatives used to visit and stay at home. By estimating the average daily water use these variables should be taken into account. Although harvested rain water can be used in drinking, cooking, washing, and bathing purposes, it is used only for drinking, cooking, and food preparation purposes in the studied household. This has been approximated due to the fact that, for livelihood, drinking as well as cooking water is thought to be more important. Based on this idea, Harun and Kabir [19] have lately investigated performance of pond sand filter (PSF) in meeting water demand for cooking and drinking purpose in southwest coastal belt of Bangladesh. Thus total water demand in the studied household is estimated to be about 6 L/person/day [20]. The number of persons in the household studied is four. So, the daily water demand for the studied household is 6 × 4 = 24 L. However, assuming five months as the longest average dry period, the storage requirement for drinking, cooking, and food preparation is 24 × 30 × 5 = 3600 L.

### 2.4. Catchment Area and Runoff Coefficient (RC)

One of the important components of RWH systems is catchment, which is used for holding rainwater. Roofs provide an ideal catchment surface for harvesting rainwater, provided that they are clean. The roof surface may be constructed of many different materials, which include, but are not limited to, concrete, tiles, galvanized corrugated iron sheets, and corrugated plastic. If a building or house with an impermeable roof, which is resistant to rain, is already in place, the catchment area is available free of charge. The studied household is a one-storied building having a concrete roof. The approximate size of the existing roof catchment area is 102 m^2^ out of which 40 m^2^ was guttered and therefore used for holding rainwater.

The collection of rain water is usually represented by a runoff coefficient (RC). The runoff coefficient for any catchment is the ratio of the volume of water that runs off a surface to the volume of rainfall that falls on the surface. A runoff coefficient of 0.8 means that 80% of the rainfall will be collected. So, the higher the runoff coefficient, the more the rain that will be collected. The roof runoff coefficient varies significantly on the basis of roof material, slope of the roof, and so forth. The roof material does not only determine the runoff coefficient; it also influences the water quality of the harvested rainwater. Painted roofs are sometimes used for rainwater collection but it is important that the paint be nontoxic and does not cause water pollution. An impermeable roof will yield a high runoff of good quality water that can be used for all domestic purposes: cooking, washing, drinking, and so forth.  Table 1 shows typical runoff coefficients for different types of catchment [5, 9]. However, a runoff coefficient of 0.8 was adopted in this study for the calculation of potential rainwater harvested from the catchment area.

### 2.5. How Much Water Can Be Harvested?

The quantity of water that runs off a roof into gutter system is usually calculated using the following equation [21]:

(1) $$\begin{array}{c} Q=\text{RC}\times R\times A, \end{array}$$

where *Q* is the quantity of water that runs off, RC is the runoff coefficient, *R* is the total rainfall (mm/y), and *A* is the roof area or the catchment area (m^2^).

Since RC is 0.8 (as adopted for this study), *R* is 1800 mm/y (for Khulna region), and *A* is 40 m^2^, the quantity of water (*Q*) that runs off (supply) is 57.6 m^3^/y (≈57600 L/y). Thus the amount of water that can be harvested is 157 L/day. However, assuming four months as the rainy season, the amount that can be harvested during this period is 157 × 30 × 4 = 18840 L, which is more than enough for storing required amount of water (3600 L) for drinking, cooking, and food preparation purposes for the studied household.

### 2.6. Components of RWH Systems

Rainwater harvesting (RWH) systems have three basic components which are (i) catchment area, (ii) supporting collection system, and (iii) storage tank.

#### 2.6.1. Catchment Area

Catchment area has already been discussed in an earlier section (Section 2.4).

#### 2.6.2. Supporting Collection System

The harvested water from a catchment area needs to be transported to the storage reservoir or tank through a system of gutters and pipes. The rule of thumb of designing gutter is that 1 cm^2^ gutter cross section would be constructed for 1 m^2^ roof surface [9, 22]. Even though several types of delivery systems exist for guttering, commonly used materials for gutters and downpipes are galvanised metal and plastic (PVC) pipes, which are readily available in local shops. Apart from that, split bamboo, which is an indigenous product, can also be used for this purpose. A well-designed gutter system can increase the longevity of a house. In this study, PVC pipe was used for making gutter and flushing system while metal net was used as screen, which was placed at the entrance of the water collection system.

#### 2.6.3. Storage Tank

To maintain the quality of harvested rain water, a simple and convenient discharge system is very important. It is essential that the first rainwater can be discharged outside the storage tank easily through flushing system. The cover of the tank should be tightly fitted to prevent evaporation and mosquito breeding and to keep spiders, lizards, and other insects from entering the tank. In this study two plastic tanks of 2000 L capacity were used. The tanks were placed on a round-shaped basement, which was approximately 3 ft above the ground. This was done in the view that water can be easily taken from the tank by opening the tap fitted with this. The tank was fitted with two outlets: one for collecting water from it and the other for discharging water from the tank during cleaning up.

### 2.7. Laboratory Tests

Sample of harvested rainwater was taken from the storage tank for the analyses of pH, turbidity, total dissolved solid, total hardness, chloride, iron, nitrate, and alkalinity. The tests were conducted at the Chemical Engineering Laboratory, Jessore University of Science and Technology, Bangladesh, and at the laboratory of Asia Arsenic Network, Jessore, Bangladesh, using standard methods (mentioned in Table 2).

## 3. Discussion and Conclusion

### 3.1. The Climate of Bangladesh

**Figure 2:**
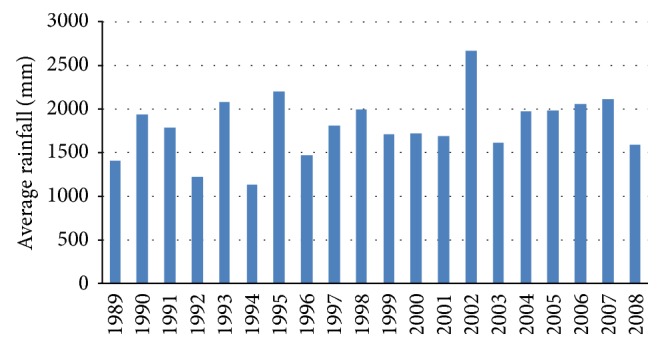
Average yearly rainfall data in Khulna district from 1989 to 2008.

**Figure 3:**
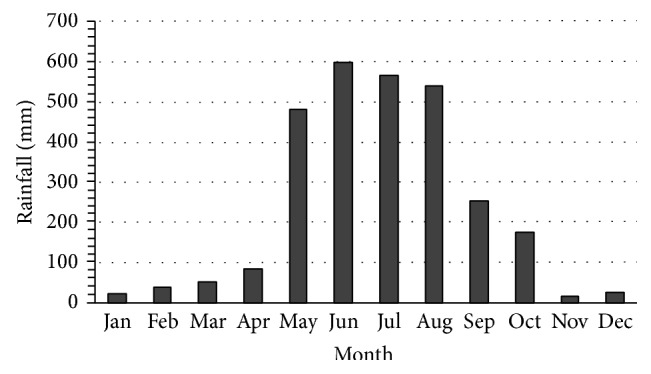
Monthly rainfall data.

Even though Bangladesh has six seasons (each season consists of two months) in literature, in fact, those are overlapped with each other. Bangladesh has a typical monsoon climate characterized by rain-bearing winds, moderately warm temperatures, and high humidity. In general, the average maximum temperature in the summer months is in the mid 30°C. Bangladesh receives heavy rainfall during the rainy season, which extends from May to September, with the peak of precipitation taking place during June, July, and August. Rain usually falls in the form of showers that can last for few minutes to several hours. The average annual rainfall under the normal climatic conditions is about 2320 mm [15] in Bangladesh while that in Khulna district is about 1800 mm. Rainfall data for a period of 20 years (from 1989 to 2008) are presented in Figure 2 while monthly rainfall data for the year of 2006 are depicted in Figure 3. Such data indicate that there is a significant amount of rainwater that can be harvested in the rainy season. On the basis of this rainfall RWH system can be effectively implemented for household usage.

### 3.2. Water Quality Considerations

Water security has been defined by WaterAid [7] as “Reliable access to water of sufficient quantity and quality for basic human needs, small-scale livelihood and local ecosystem services, coupled with a well managed risk of water-related disasters.” Therefore, a question arises: how safe is the rain water? The safety of water can be determined at the household level by people's perception and by laboratory analyses. Since rainwater does not contain any minerals and does not carry any taste, it is not widely accepted as drinking water. Most of the existing rainwater tanks are generally not tested for water quality; therefore, householders have no knowledge of true water quality, but rather they have only the perception of water quality. The quality of rain water depends on several issues, which include, but are not limited to, the following: location, rainfall intensity, number of dry days preceding a rainfall, and rainwater collection system as well as storage method. The harvested and stored rainwater may not always meet the WHO standards [23, 24] as it is usually free from minerals and salt.

### 3.3. Physical and Chemical Tests of Harvested Rainwater

Rural areas are usually far apart from industrial pollution [25]. The current study area is similar to this location. As a result the rainwater in the study area is anticipated to be clean except for some dissolved gases. However, the harvested rainwater was tested and the results are depicted in Table 2 alongside Bangladesh national standards as well as WHO standards. The result shows that pH of the harvested rainwater is within acceptable limits of both Bangladesh national standards and WHO standards. The result also shows that there is almost no dissolved solid. Other tested parameters are of very good conformity with standard values. However, the presence of chloride ion in harvested rainwater is because rainwater acquires its chloride content from the large bodies of salt water of the Bay of Bengal. Moreover, when the ocean evaporates, some anions travel with the water vapor [26]. Total dissolved solid (TDS) in harvested rainwater is found to be significantly low compared to the drinking water standards. This can be attributed to the fact that most of the pollutants in atmosphere are washed away after 5–20 minutes of rain depending on the intensity of rainfall. The result of the quality test of the sample indicated that harvested rainwater does not contain anything harmful for drinking. Even though Shittu et al. [5] suggested that addition of sodium hypochlorite solution would enhance the quality of harvested water, in this current work the analyses report showed that without addition of any such chemicals the harvested rain water quality remains within the standards. This means that the harvested rainwater contained in the storage tank is of good quality and suitable for domestic use. However, after building up the rainwater harvesting system, it is important to monitor and test the water quality on a regular seasonal basis. It is, however, recommended that the stored water should be chlorinated once in every rainy season. It is further recommended that catchment area and supporting rainwater collection system must be cleaned before the start of the rainy season.

## 4. Conclusion

This study evaluated the feasibility of rainwater harvesting and its domestic usage in a very remote locality of Bangladesh where there is severe scarcity of drinking water. A rainwater harvesting system for a small household was constructed by using commodities and resources available in local markets and was found to be very much effective as well as viable. It was found that the amount of harvested and stored rainwater could be utilized not only in rainy season but also over the whole dry periods of the year for the studied household. Although the system generally poses a potential pollution problem with bacteria, the other physicochemical characteristics of harvested rainwater showed that those were in good accordance with the standards of Bangladesh and those of WHO.

There are so many misconceptions in the world and rainwater is no exception. When people think about rainwater, they often erroneously think that it contains pollutants. But the truth is that rainwater is extremely clean and safe if the location is in rural area where highway traffic and urbanization are far-reaching. So in such area if rainwater can be collected and stored in a proper and scientific manner, management of water resources would enter a new era. Since the discussed roof harvesting technology does not have any harmful effect on the environment, rainwater harvesting seems to be a beneficial and sustainable method. Therefore, advocacy for the adoption of rainwater would certainly lead to a reduction of problems related to water shortage in monsoon-prone country like Bangladesh.

## Figures and Tables

**Table 1:** Runoff coefficients for traditional roofing materials.

Type	Runoff coefficient
Galvanized iron sheet	>0.9
Corrugated metal sheet	0.7–0.9
Tiles	0.8-0.9
Concrete	0.6–0.8
Brick pavement	0.5-0.6
Rocky natural catchment	0.2–0.5
Soil with slope	0.0–0.3
Green area	0.05–0.1

**Table 2:** Physicochemical characteristics of harvested rainwater.

Parameter (unit)	Used methods	Harvested rainwater	Bangladesh drinking water quality standards	WHO standards
Chloride (mg/L)	Mohr's	6.50	150–600	200–300
Iron (mg/L)	Colorimetric	0.11	0.30–1.00	0.3
Nitrate (mg/L)	Cd reduction	0.40	50	50
pH	Membrane electrode	6.72	6.5–8.5	6.8–7.3
Turbidity (NTU)	Spectrophotometric	<1.00	6.00	5.0
TDS (mg/L)	Conductivity	12	500–1000	1000
Total hardness (mg/L)	EDTA	<10	500	40
Alkalinity (mg/L)	Mohr's	50	50	40
